# A novel diagnostic system to evaluate epidermal growth factor receptor impact as a prognostic and therapeutic indicator for lung adenocarcinoma

**DOI:** 10.1038/s41598-020-63200-7

**Published:** 2020-04-10

**Authors:** Kazuya Takakuwa, Kaoru Mogushi, Min Han, Tomoaki Fujii, Masaki Hosoya, Arina Yamanami, Tomomi Akita, Chikamasa Yamashita, Tetsu Hayashida, Shunsuke Kato, Shigeo Yamaguchi

**Affiliations:** 1 Department of Clinical Oncology, Juntendo University Graduate School of Medicine, Hongo, Bunkyo-ku, Tokyo Japan; 2 Department of Pharmaceutics and Drug Delivery, Faculty of Pharmaceutical Sciences, Tokyo University of Science, Yamazaki, Noda, Chiba Japan; 3 Department of Cancer Genome Research, Sasaki Institute, Sasaki Foundation, Kandasurugadai, Chiyoda-ku, Tokyo Japan; 4 International School of the Sacred Heart, Shibuya-Ku, Tokyo Japan; 5 Department of Surgery, Keio University School of Medicine, Shinanomachi 35, Shinjuku-ku, Tokyo Japan

**Keywords:** Non-small-cell lung cancer, Cellular signalling networks

## Abstract

Many driver pathways for cancer cell proliferation have been reported. Driver pathway activation is often evaluated based on a single hotspot mutation such as EGFR L858R. However, because of complex intratumoral networks, the impact of a driver pathway cannot be predicted based on only a single gene mutation. Here, we developed a novel diagnostic system named the “EGFR impact score” which is based on multiplex mRNA expression profiles, which can predict the impact of the EGFR pathway in lung cancer cells and the effect of EGFR-tyrosine kinase inhibitors on malignancy. The EGFR impact score indicated robust predictive power for the prognosis of early-stage lung cancer because this score can evaluate the impact of the EGFR pathway on the tumor and genomic instability. Additionally, the molecular features of the poor prognostic group resembled those of biomarkers associated with immune checkpoint inhibitors. The EGFR impact score is a novel prognostic and therapeutic indicator for lung adenocarcinoma.

## Introduction

EGFR mutations are frequently reported in lung adenocarcinoma^1,2^. The missense mutation L858R and exon 19 deletion is a well-known hotspot mutation in EGFR^3,4^. EGFR hotspot mutations activate the EGFR pathway and promote tumor cell proliferation^5^. Conversely, next-generation sequencing has revealed the presence of many variants of uncertain significance (VUSs) in the EGFR gene^6^. To date, a method for evaluating these VUSs has not been established.

In the clinical setting, EGFR hotspot mutations are considered surrogate markers of tyrosine kinase inhibitor (TKI) treatment. However, approximately 30% of EGFR-mutant cancers exhibit resistance to TKI treatment without well-known secondary EGFR mutations such as T790M^7^. Some papers reported that these TKI-resistant tumors depend on both EGFR signaling and multiple driver pathways for survival^8,9^. Contrarily, the presence of tumors that respond to TKIs despite not carrying EGFR structural mutations has been reported^10,11^. These results indicated the existence of EGFR pathway activation in the absence of EGFR structural mutations. These findings illustrated the limitation of using single gene mutations to predict the effects on EGFR network signaling. We hypothesized that mRNA expression profiles can predict the complicated EGFR network status more comprehensively than the DNA structural status because of central dogma. Based on this hypothesis, this study first aimed to develop a predictive model of responsiveness to TKI based on mRNA expression profiles.

It is controversial whether EGFR mutation is a prognostic factor in early-stage lung adenocarcinoma^12–16^. Therefore, we clarified whether accurate evaluation of EGFR networks can predict the prognosis of early-stage lung adenocarcinoma. In this study, we evaluated EGFR networks via multiplex gene expression profiling. Several prognostic prediction models based on expression profiles have been developed for lung cancer^17–21^. Some expression profiles are currently in clinical use and are commercially available^22^. However, these expression profiles do not influence treatment decision-making. Considering these situations, the second purpose of this study was to clarify whether EGFR network expression profiles can be used to predict the prognosis of early-stage lung adenocarcinoma. Furthermore, to develop new treatment strategies for the poor prognostic group identified using our new system, we clarified the molecular biological features of lung cancer using multi-omics data.

## Results

### Establishment of the EGFR impact score by analyzing a publicly available dataset

**Figure 1:**
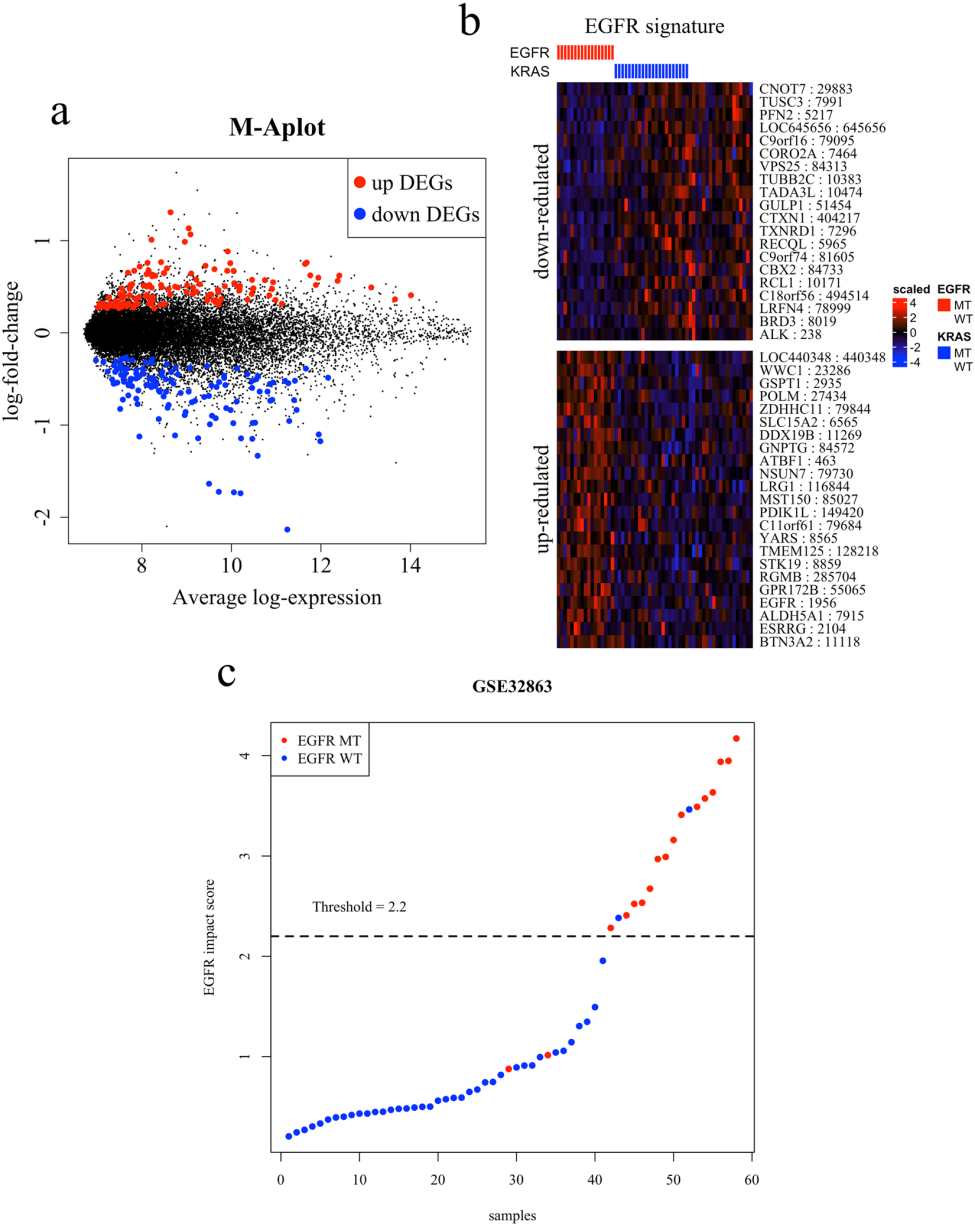
(**a**) MA plot. Each dot denotes upregulated (red) and downregulated (blue) genes among patients with EGFR mutations in the GSE32863 dataset. (**b**) Heatmap of the EGFR signature in GSE32863. The rows of the heatmap represent gene symbols and Entrez IDs, and samples are presented in columns. The labels at the top denote the structural status of the EGFR and KRAS genes. (**c**) EGFR impact score in the GSE32863 dataset. The x-axis shows samples, and the y-axis shows the EGFR impact score. MT, mutant; WT, wild-type.

We identified 254 differently expressed genes (DEGs) in EGFR-mutant cancers in the GSE32863 dataset (Fig. 1a). We analyzed 58 patients in this dataset, 17 of whom had an EGFR mutation. Of the 254 DEGs, 42 were selected using the random forest algorithm. Of these selected DEGs, 23 were upregulated and 19 were downregulated in EGFR-mutant cancers. These gene sets were designated as the upregulated EGFR signature and downregulated EGFR signature, respectively. The ALK gene was added to the downregulated EGFR signature group (Fig. 1b). Using the expression profile of each EGFR signature, we calculated the EGFR impact score. We conducted receiver operating characteristic (ROC) analysis to determine the threshold of the EGFR impact score (Supplemental Fig. 1). Patients with an EGFR impact score higher than 2.2 comprised the EI-H-type group, and those with an EGFR impact score lower than 2.2 comprised the EI-L-type group (Fig. 1c).

### Prediction of responsiveness to TKI treatment based on the EGFR impact score

**Figure 2:**
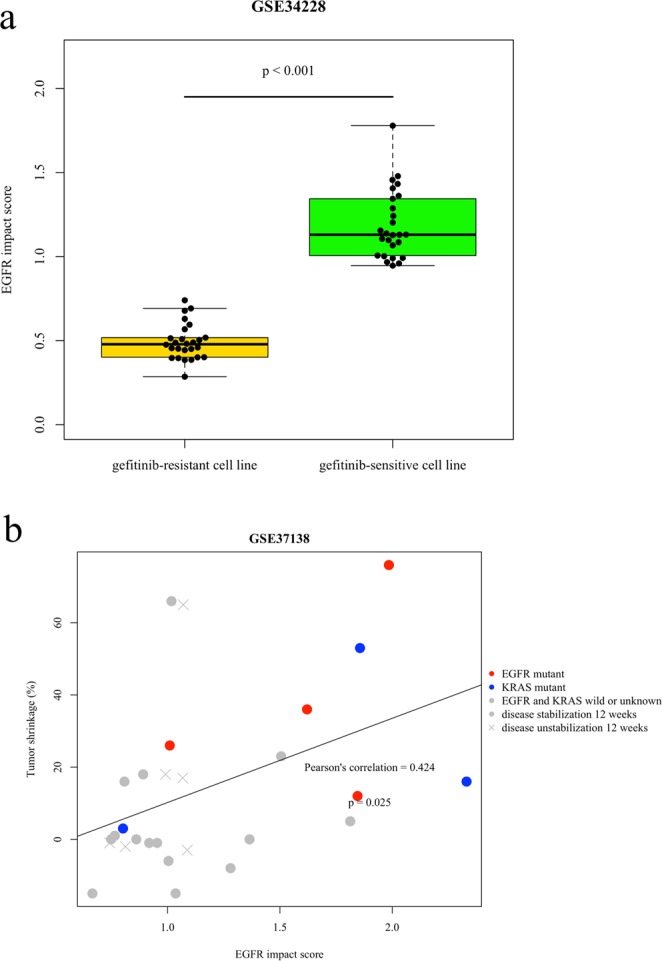
(**a**) EGFR impact score in the GSE34228 dataset. The boxplot in yellow shows the EGFR impact score in gefitinib-resistant cell lines, and the boxplot in green shows gefitinib-sensitive cell lines. (**b**) Association between the EGFR impact score and tumor shrinkage rate in GSE37138. Each dot shows patients with mutant EGFR (red), mutant KRAS (blue), and other (grey).

We analyzed GSE34228 data (n = 52) to clarify whether the EGFR impact score can predict responsiveness to TKIs. The GSE34228 dataset contains expression data from both gefitinib-sensitive and gefitinib-resistant cell lines. This dataset uses the PC-9 cell line, which carries the delE746-A750 mutation in EGFR^23^. The gefitinib-resistant cell line was created by exposing PC-9 cells to long-term culture with gefitinib. We calculated the EGFR impact score in these cell lines. The EGFR impact score of the gefitinib-resistant cell line was significantly lower than that of the gefitinib-sensitive cell line (Fig. 2a). Moreover, we analyzed CCLE (Cancer Cell Line Encyclopedia) and GDSC (Genomics of Drug Sensitivity in Cancer) data sets. CCLE and GDSC datasets have information of both IC50 of gefitinib and expression profile data in EGFR mutant cell lines. We circulated the EGFR impact score of each cell line. The EGFR impact score was clearly associated with IC50 of gefitinib in both CCLE and GDSC data sets (Supplementary Fig. 2a,b). Notably, In the GDSC data set, the EGFR impact score was clearly correlated with IC50 of gefitinib. Furthermore, we analyzed GSE37138 data to clarify whether the EGFR impact score can predict responsiveness to TKIs in clinical specimens. In GSE37138 data, patients with non-squamous non-small cell lung cancer (NSCLC) were treated with erlotinib and bevacizumab. The tumor shrinkage rate was assessed in all patients 12 weeks after treatment. Of these patients, four patients had EGFR mutations, and three patients had KRAS mutations. The dataset included patients with high EGFR impact scores and good responsiveness to treatment despite not carrying EGFR mutations (Fig. 2b). In the GSE37138 dataset, Although Fig. 2b necessitate the recruitment of more patients, EGFR impact score tends to predict responsiveness of TKI treatment in clinical samples.

### Diagnosis of patients with VUSs using the EGFR impact score

**Figure 3:**
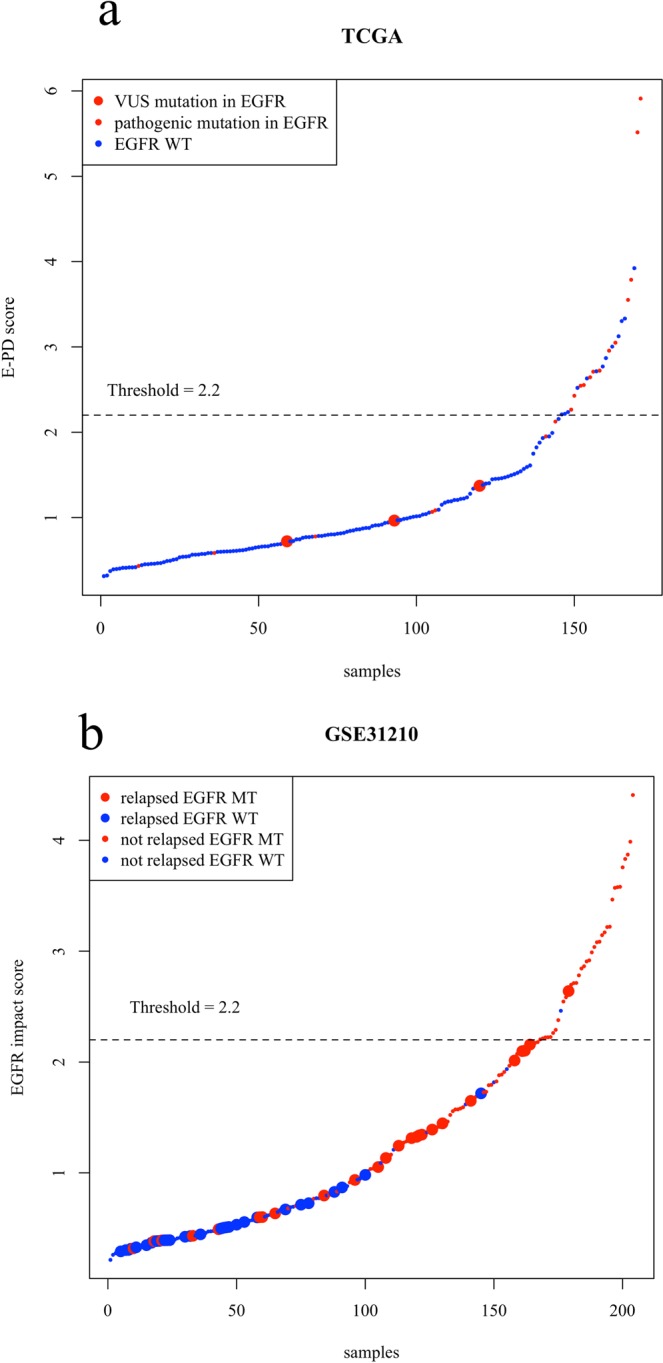
(**a**) EGFR impact score in the TCGA dataset. The x-axis shows samples, and the y-axis shows the EGFR impact score. Larger dots denote patients with VUSs in EGFR. (**b**) EGFR impact score in the GSE31210 dataset. The x-axis shows samples, and the y-axis shows the EGFR impact score. Larger dots denote patients with recurrence. VUS, variant of unknown significance; WT, wild-type; MT, mutant.

We analyzed TCGA data to clarify the classification of patients with VUSs in the EGFR gene using the EGFR impact score. Patients with early-stage lung adenocarcinoma (n = 171) were selected, and of these patients, 24 had EGFR mutations. Six patients carried exon 19 deletions, six patients carried the L858R mutation, three patients had VUSs and the remaining patients had other mutations in EGFR. Sixty-one percent of patients with pathogenic mutations were diagnosed with EI-H-type cancer, and the remaining patients were diagnosed with EI-L-type cancer. All patients with VUSs were diagnosed with EI-L-type cancer (Fig. 3a).

### Prediction of prognosis using the EGFR impact score

**Figure 4:**
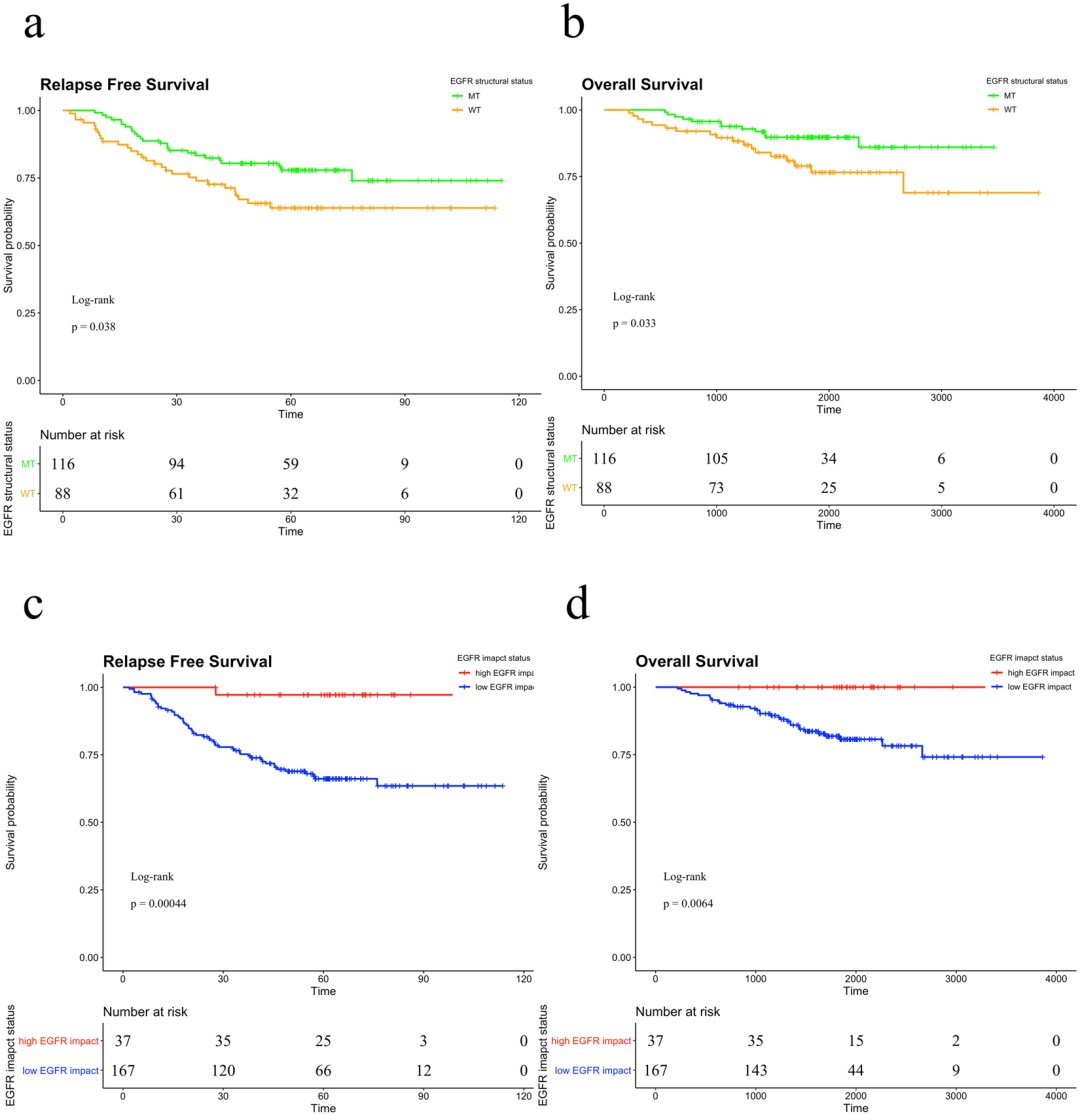
(**a,b**) Recurrence-free survival (RFS) and overall survival (OS) curves, respectively, according to the EGFR status in the GSE31210 dataset. The green line denotes patients with EGFR structural mutations, and the orange line denotes patients with wild-type EGFR. (**c,d**) RFS and OS curves, respectively, according to the EGFR impact status in the GSE31210 dataset. The red line denotes the EI-H-type group, and the blue line shows the EI-L-type group. MT, mutant; WT, wild-type.

**Figure 5:**
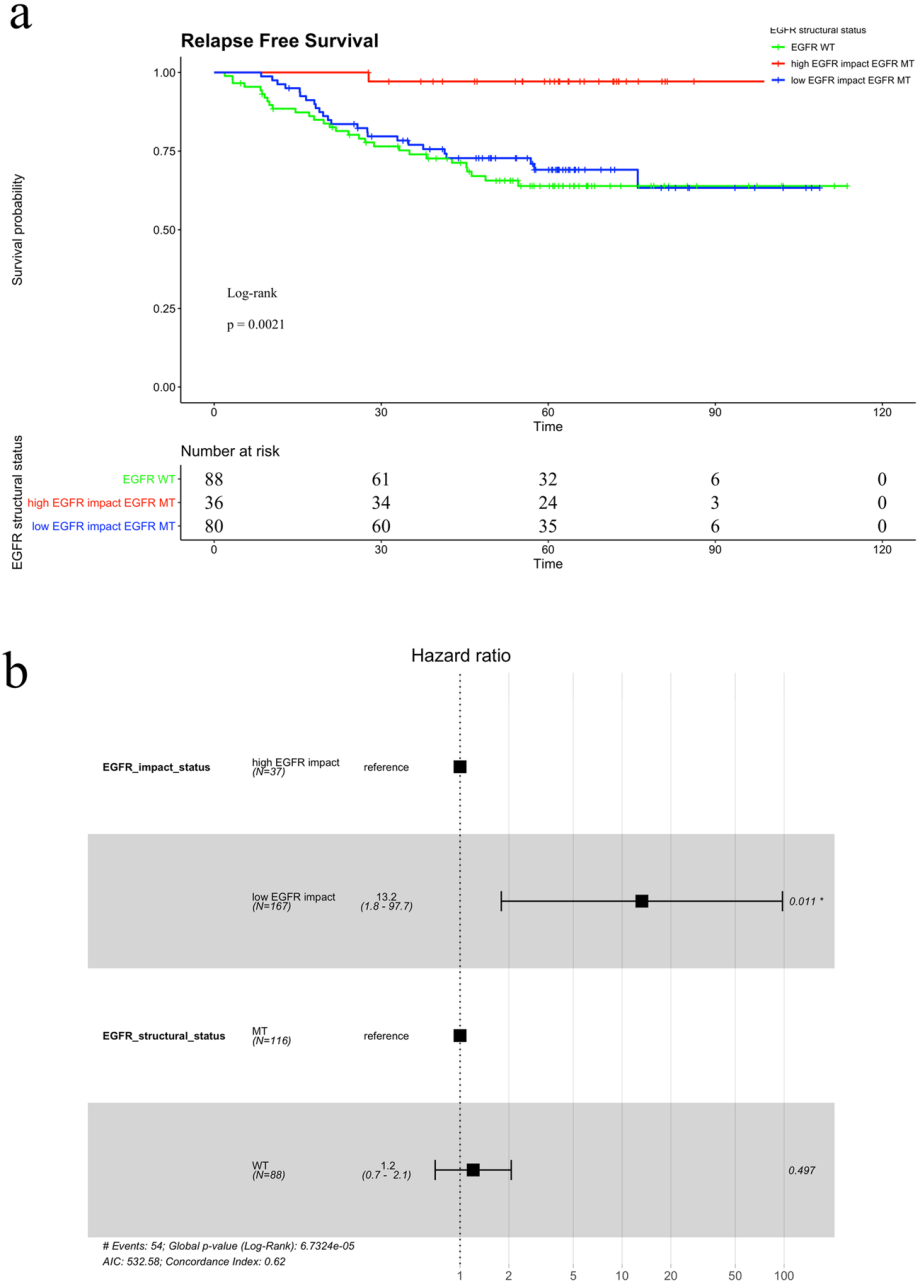
**(a**) Recurrence-free survival (RFS) curves according to the EGFR structural status and EGFR impact status in the GSE31210 dataset. The different coloured lines denote the EGFR-mutant EI-H-type (red), EGFR-mutant EI-L-type (blue) and EGFR wild type group (green). (**b**) Multivariate analysis by the EGFR structural status and EGFR impact score regarding RFS in the GSE31210 dataset using the Cox hazard model. MT, mutant; WT, wild-type.

We analyzed the GSE31210 dataset to clarify whether the EGFR impact score can predict the prognosis of early-stage lung adenocarcinoma more accurately than EGFR structural mutations. In this dataset, patients who did not receive adjuvant therapy (n = 204) were selected, and of these patients, 116 had EGFR mutations. Thirty-one percent of patients with EGFR mutations were diagnosed with EI-H-type cancer, and the remaining patients were diagnosed with EI-L-type cancer (Fig. 3b). We compared recurrence-free survival (RFS) and overall survival (OS) between patients with wild-type EGFR and EGFR structural mutations. Patients with wild-type EGFR had poorer RFS and OS than patients carrying EGFR mutations (Fig. 4a,b). Similarly, we compared the prognosis of the EI-H-type and EI-L-type groups. The EI-L-type group also had poorer RFS and OS than the EI-H-type group (Fig. 4c,d). Among patients with EGFR-mutant cancer, the EI-L-type group had poorer prognosis than the EI-H-type group (Fig. 5a). Multivariate analysis using the Cox proportional hazard model indicated that the EGFR impact score can predict prognosis more accurately than the EGFR structural status (Fig. 5b). In addition to GSE31210, we analyzed GSE11969 data set which has early stage lung adenocarcinoma patients with EGFR mutation. In GSE11969, EI-L type tended to have poorer OS than EI-H type (Supplementary Fig. 3).

### Biological features of the EI-H-type and EI-L-type groups

**Figure 6:**
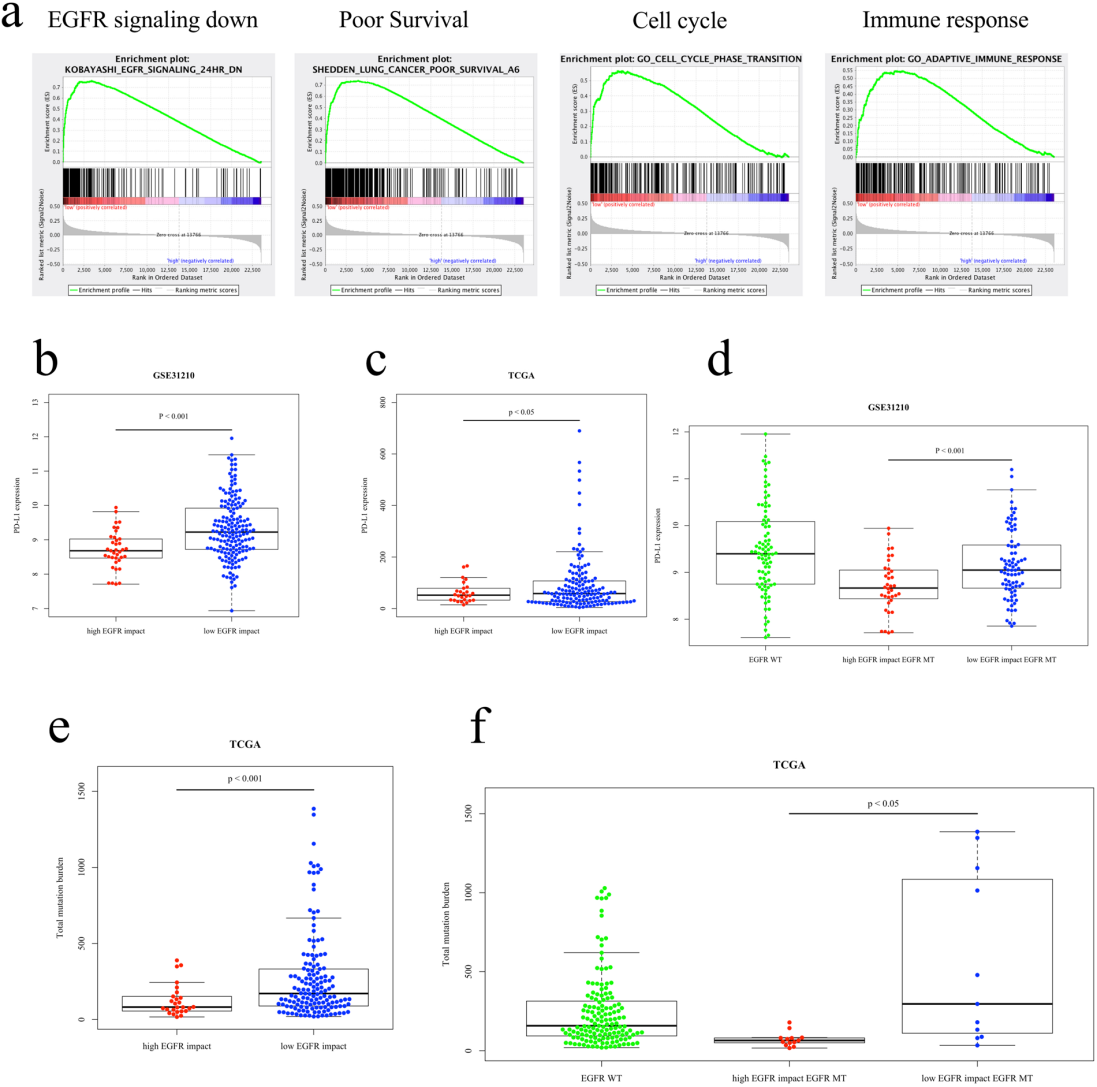
(**a**) Results of gene set enrichment analysis using the GSE31210 dataset. These gene sets are enriched regarding patients with EI-L-type cancer and EGFR mutations. (**b**) Microarray data of PD-L1 expression in the GSE31210 dataset. (**c**) RNA sequence data of PD-L1 expression levels of PD-L1 expression levels in the EGFR-mutant EI-H-type (red), EGFR-mutant EI-L-type (navy), wild-type EGFR EI-H-type (blue), and wild-type EGFR EI-L-type groups (yellow). (**d**) Microarray data of PD-L1 expression in the EGFR-mutant EI-H-type (red), EGFR-mutant EI-L-type (blue), EGFR wild type groups (green) in the GSE31210 dataset.. (**e**) Total mutation burden of the exome sequence in the TCGA dataset. Red dots denote the mutation burden in the EI-H-type group, and blue dots denote the mutation burden in the EI-L-type group. (**f**) Total mutation burden of the exome sequence in the EGFR-mutant EI-H-type (red), EGFR-mutant EI-L-type (blue), EGFR wild type groups (green) in the TCGA dataset.

Using GSE31210 data, we compared biological molecular features between the EI-H-type and EI-L-type groups among patients with EGFR mutations via gene set enrichment analysis (GSEA). The GSEA results indicated that gene sets related to “EGFR signaling down,” “poor survival,” “the cell cycle,” and “immune response” were enriched in the EI-L-type group (Fig. 6a), and gene sets related to “good survival” were enriched in the EI-H-type group. We compared PD-L1 expression levels and somatic mutation burden between the EI-H-type and EI-L-type groups. In the GSE31210 and TCGA datasets, PD-L1 levels were significantly higher in the EI-L-type group than in the EI-H-type group (Fig. 6b,c). Restricting the analysis to only patients with EGFR mutations, PD-L1 expression was significantly higher in the EI-L-type group than in the EI-H-type group in the GSE31210 dataset (Fig. 6d). In the TCGA dataset, the EI-L-type group had a significantly higher total mutation burden than the EI-H type group (Fig. 6e). Among patients with EGFR mutations, the total mutation burden was significantly higher in the EI-L-type group than in the EI-H-type group (Fig. 6f).

## Discussion

We developed a novel diagnostic system to predict the EGFR network status comprehensively based on gene expression profiles, which we named the EGFR impact score. Using TCGA data, the EGFR impact score distinguished pathogenic mutations in the EGFR gene from VUSs. Whether these VUSs are pathogenic is unclear because we did not conduct forward genetic experiments using cell or animal models. However, the result that the EGFR impact score can distinguish hotspot mutations from VUSs in the EGFR gene means the strategy can potentially predict the functional abnormality of VUSs that have been unreported as pathogenic mutations. In the analysis of the cell line model, the EGFR impact score identified cells that are resistant to gefitinib despite carrying hotspot EGFR mutations. This result illustrates that the EGFR impact score can categories the same group classified by the EGFR structural status into more detailed groups based on differences in EGFR pathway dependency. Nakata *et al*., who established a gefitinib-resistant cell line, indeed reported activation of oncogenic pathways in addition to the EGFR pathway in the cell line^24^. In an analysis of clinical specimens, the EGFR impact score also predicted responsiveness to TKIs, although there is a limitation that patients were not treated with erlotinib alone. The EGFR impact score tends to predict the responsiveness to TKI treatment in clinical samples, indicating that this score can accurately predict EGFR pathway dependency irrespective of the presence of EGFR structural mutations. These results demonstrate that the EGFR impact score can predict responsiveness to TKIs by evaluating EGFR pathway dependency.

In addition, Although follow-up time is short, the EGFR impact score also predicted prognosis in early-stage lung adenocarcinoma. Yixin *et al*. reported that genomic instability is caused by defects in DNA damage checkpoints, DNA repair, and mitotic checkpoints^25^. Our results found that EI-L-type lung adenocarcinoma has a high mutation burden, suggesting that this type has genomic instability because of abnormality of DNA repair. Several studies reported that tumors with genomic instability have a poor prognosis^26,27^. Accordingly, the EGFR impact score is a novel diagnostic system that can evaluate genomic instability in addition to EGFR pathway dependency. The EGFR signature indeed includes genes associated with DNA repair. A prior report identified a subgroup of patients with EGFR-mutant lung adenocarcinoma featuring genomic instability^28^. Our results indicated that one reason why findings regarding the relationship of the EGFR structural status with the prognosis of early-stage lung adenocarcinoma differed in past reports is that genomic instability cannot be evaluated using the EGFR structural status alone. As the EGFR impact score can evaluate genomic instability, this method accurately identified a poor prognostic group that could not be classified using the EGRF structural status alone. In summary, the EI-H-type group has pure EGFR pathway dependency, whereas the EI-L-type group features genomic instability. An EGFR mutant tumor without pure EGFR dependency is a tumor with genomic instability, as a result of which the prognosis for this patient group is poor. The EGFR impact score is a new diagnostic system that can evaluate two major events in oncogenesis, namely driver gene and DNA repair defects.

The EI-L-type group exhibited a high mutation burden and high PD-L1 expression. These features are consistent with biomarkers associated with immune checkpoint inhibitors^29,30^. Patients with driver mutations such as EGFR mutations display a low mutation burden^31^, and the efficacy of immune checkpoint inhibitor in such patients is controversial^32^. The EGFR impact score identified patients with high PD-L1 expression and a high mutation burden. Tumors with high immunogenicity based on high genomic instability and a high mutation burden will likely respond to treatment with an immune checkpoint inhibitor. Accordingly, although EI-L-type lung adenocarcinoma is expected to exhibit resistance to TKIs and a poor prognosis, patients in this group can potentially respond to immune checkpoint inhibitors. Although the low EGFR impact group may respond to an immune checkpoint inhibitor, further study of the efficacy of immune checkpoint inhibitors is required.

We developed a novel diagnostic system that can evaluate EGFR networks. The EGFR impact score can predict the responsiveness to TKI treatment in patients with EGFR mutant lung adenocarcinoma. Additionally, this diagnostic system can predict the prognosis of early-stage lung adenocarcinoma because it reflects genomic instability. Furthermore, immune checkpoint inhibition could represent an alternative treatment strategy for patients predicted to have poor prognoses using the EGFR impact system. In summary, the EGFR impact score is a novel diagnostic system with the potential to change standard treatment strategies.

## Methods

### Publicly available datasets

The gene expression datasets GSE32863, GSE37138, GSE34228, GSE31210, and GSE11969 were downloaded from the National Center for Biotechnology Information Gene Expression Omnibus database^24,33–37^. The TCGA dataset (Lung Adenocarcinoma in TCGA Provisional) and CCLE (Cancer Cell Line Encyclopedia) were downloaded via cBioportal^38,39^. GDSC (Genomics of Drug Sensitivity in Cancer) was downloaded via GDSC homepage^40,41^. Among these datasets, the expression data of GSE31210 and GSE37138 were calculated using the RMA algorithm from CEL files using “R package Affy Ver. 1.58.0” and “R package oligo Ver. 1.46.0,” respectively^42,43^. Other downloaded expression data were normalized data. All expression profiles were transformed into the logarithmic scale for ssGSEA. In the GSE31210 dataset, the EGFR mutations are defined as exon 19 del and L858R. In the TCGA dataset, the pathogenicity of the EGFR mutation is defined by OncoKB^44^.

### Determination of DEGs in EGFR-mutant lung adenocarcinoma

Using expression data from the GSE32863 dataset (n = 58, tumor samples), we identified DEGs between EGFR-mutant and wild-type samples. As the KRAS gene is a downstream molecule of the EGFR signaling pathway, we excluded tumor samples with KRAS mutations from analyses of DEGs, which were identified using “R package limma Ver. 3.36.5”^45^. DEGs were defined as follows: genes with p < 0.01 and fold change >1.2 or <1/1.2. The variable importance of DEGs was calculated via the random forest algorithm using “R package RandomForest Ver. 4.6.14^46^, and excluding genes with a variable importance of 0, genes with variable importance exceeding the third quartile were selected. We considered these DEGs and the ALK gene as EGFR signatures. Accordingly, EGFR signatures that were upregulated in samples with EGFR mutations were referred to as upregulated EGFR signatures, and the remaining DEGs were referred to as downregulated EGFR signatures.

### Diagnostic algorithm of the EGFR score classification

We classified early-stage lung adenocarcinoma as EI-H-type or EI-L-type based on the EGFR impact score. The EGFR impact score was calculated on the basis of ssGSEA using the expression profile of the EGFR signature. The EGFR impact score was calculated as follows:

$$\frac{(\text{ssGSEA}\,\text{score}\,\text{of}\,\text{upregulated}\,\text{EGFR}\,\text{signature}+1)}{(\text{ssGSEA}\,\text{score}\,\text{of}\,\text{downregulated}\,\text{EGFR}\,\text{signature}+1)}$$

ssGSEA was performed using “R package GSVA Ver. 1.30.0”^47^. The threshold of the EGFR impact score was determined via ROC analysis, which was performed using “R package ROCR Ver. 1.0.7”^48^.

### Evaluation of responsiveness to TKIs

The authors of the GSE34228 dataset (n = 52, untreated samples) examined the differences between gefitinib-sensitive and gefitinib-resistant cell lines and established a gefitinib-resistant cell line from gefitinib-sensitive PC9 cells via long-term exposure to the drug. We compared the EGFR impact score between gefitinib-sensitive and gefitinib-resistant cell lines. Welch’s *t*-test was used to evaluate the difference in the EGFR impact score. EGFR mutant lung adenocarcinoma cell lines were analyzed CCLE (n = 6) and GDSC (n = 8). Correlation between EGFR impact score and IC50 of gefitinib was evaluated using Pearson’s product moment correlation coefficient. Furthermore, we analyzed GSE37138 data. In these data, patients with non-squamous NSCLC were treated with erlotinib and bevacizumab. The patients whose tumor shrinkage rates were available (n = 28) were selected, and the EGFR impact score of these patients was calculated. The association between the EGFR impact score and the tumor shrinkage rate was evaluated using Pearson’s product moment correlation coefficient.

### Evaluation of VUSs

We classified patients with early-stage lung adenocarcinoma in the TCGA dataset (n = 171) using the EGFR impact score.

### Evaluation of RFS and OS

The log-rank test was used to evaluate the differences in RFS between the EGFR-mutant and wild-type groups and the differences in OS between the EI-H-type and EI-L-type groups in the GSE31210 dataset. Multivariate analysis using the Cox proportional hazard model was conducted to compare the prediction of recurrence based on the EGFR structural status and EGFR impact score. In addition to GSE31210, GSE11969 was analyzed. Only patients with EGFR mutant lung adenocarcinoma were used for evaluating differences in OS between the EI-H-type and EI-L-type groups (n = 32).

### GSEA

GSEA was performed using the javaGSEA Desktop Application between the EI-H-type and EI-L-type groups among patients with EGFR-mutant cancer^49,50^. We used the gene sets of the predefined C2 and C5 categories^51^.

### Evaluation of PD-L1 expression levels

The PD-L1 expression levels in the EI-H-type and EI-L-type groups were evaluated using Welch’s t-test with GSE31210 and TCGA data.

### Evaluation of tumor mutation burden

Non-synonymous mutations were summed and defined as the somatic mutation burden in the TCGA dataset. Welch’s *t*-test was used to compare the mutation burden between the EI-H-type and EI-L-type groups.

### Statistical analysis

All analyses were performed using the programming language R Ver. 3.5.0.

## Supplementary information

Supplementary Figure 1.

Supplementary Figure 2.

Supplementary Figure 3.

Supplementary information.
