# Supplementary figures and images for: A novel diagnostic system to evaluate epidermal growth factor receptor impact as a prognostic and therapeutic indicator for lung adenocarcinoma
Source: Sci Rep. 2020 Apr 10;10:6214. doi: 10.1038/s41598-020-63200-7 (PMC7148318; doi:10.1038/s41598-020-63200-7)

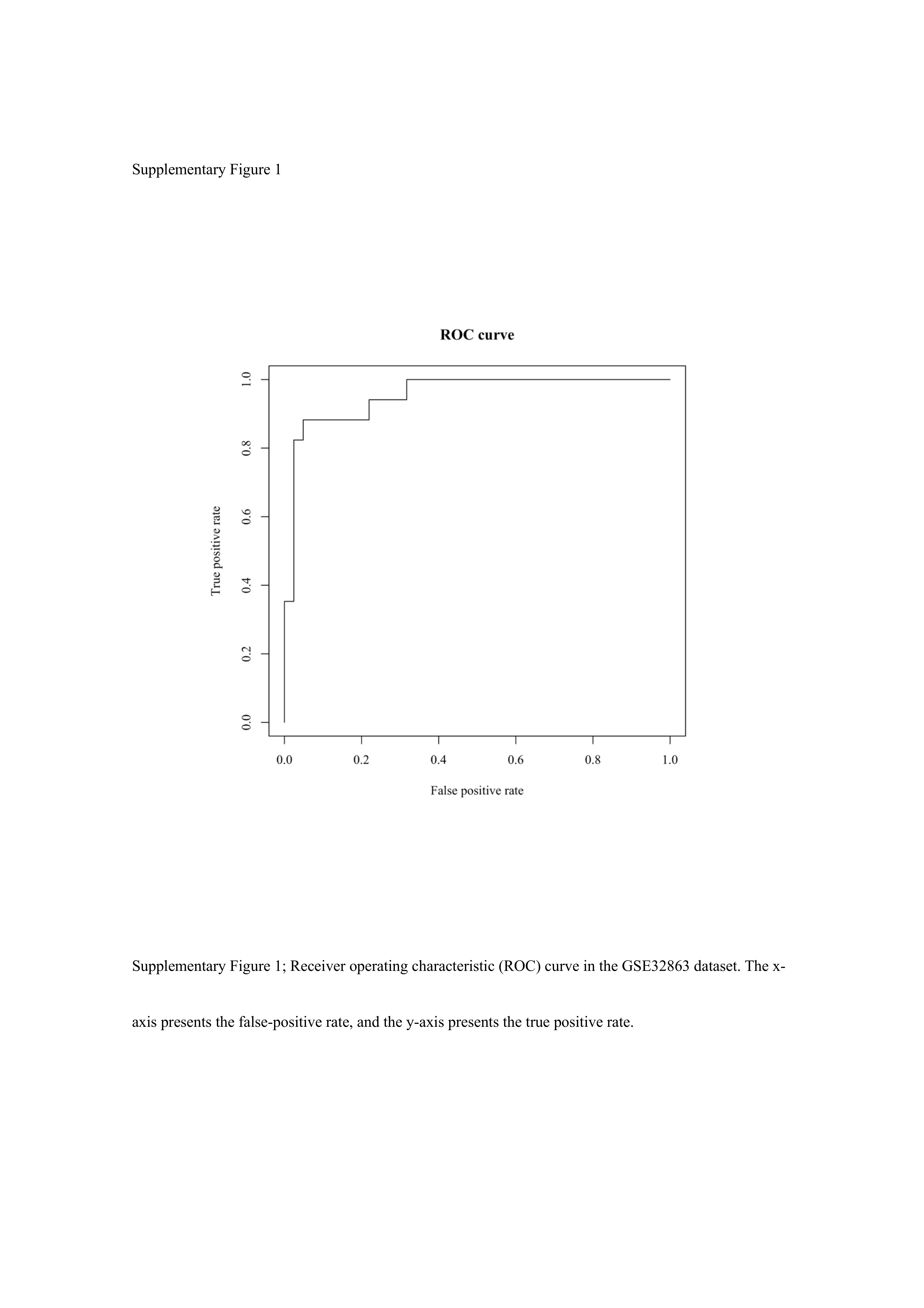

Supplement: Supplementary file 1 — Supplementary Figure 1. [file 41598_2020_63200_MOESM1_ESM.jpg]

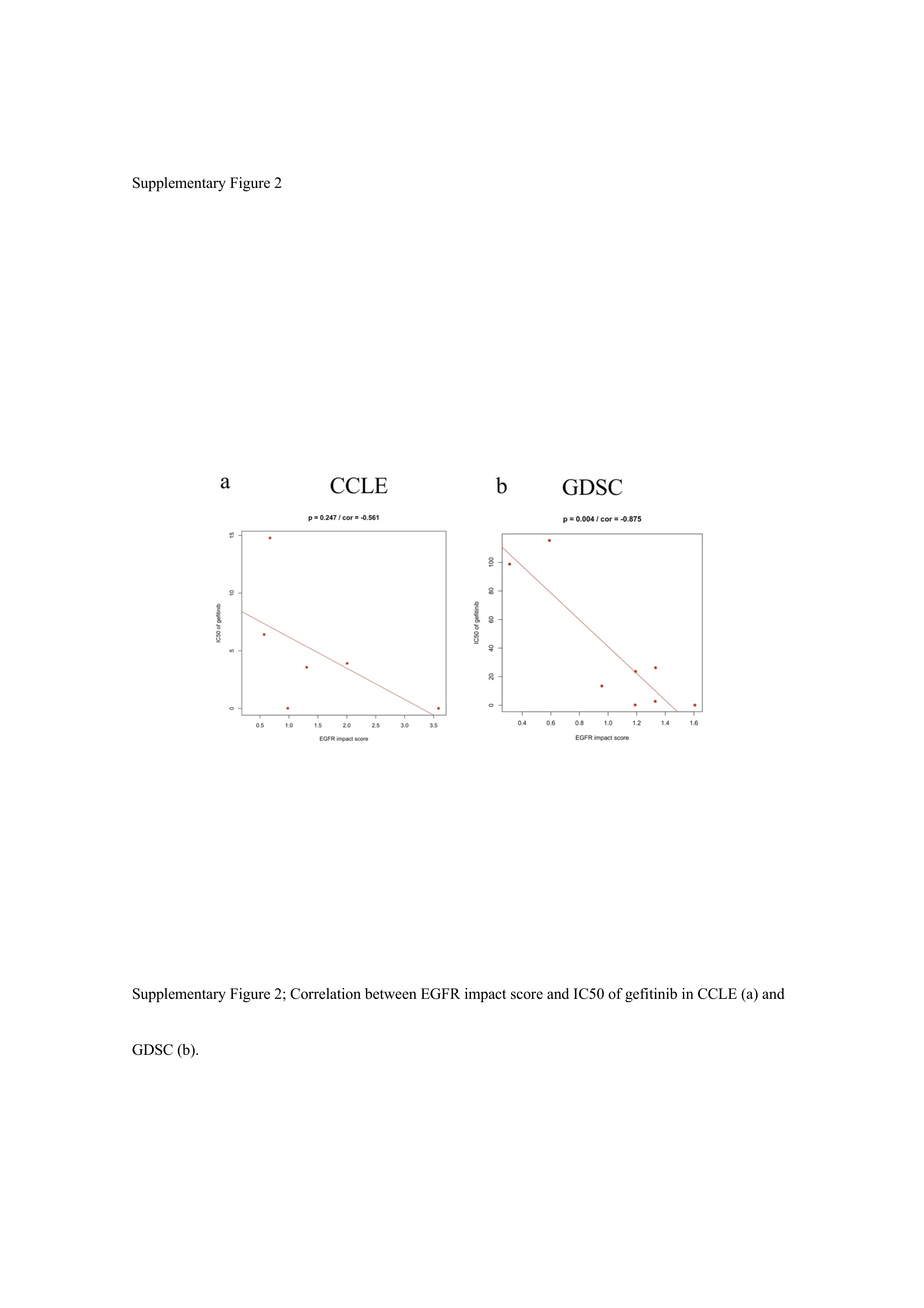

Supplement: Supplementary file 2 — Supplementary Figure 2. [file 41598_2020_63200_MOESM2_ESM.jpg]

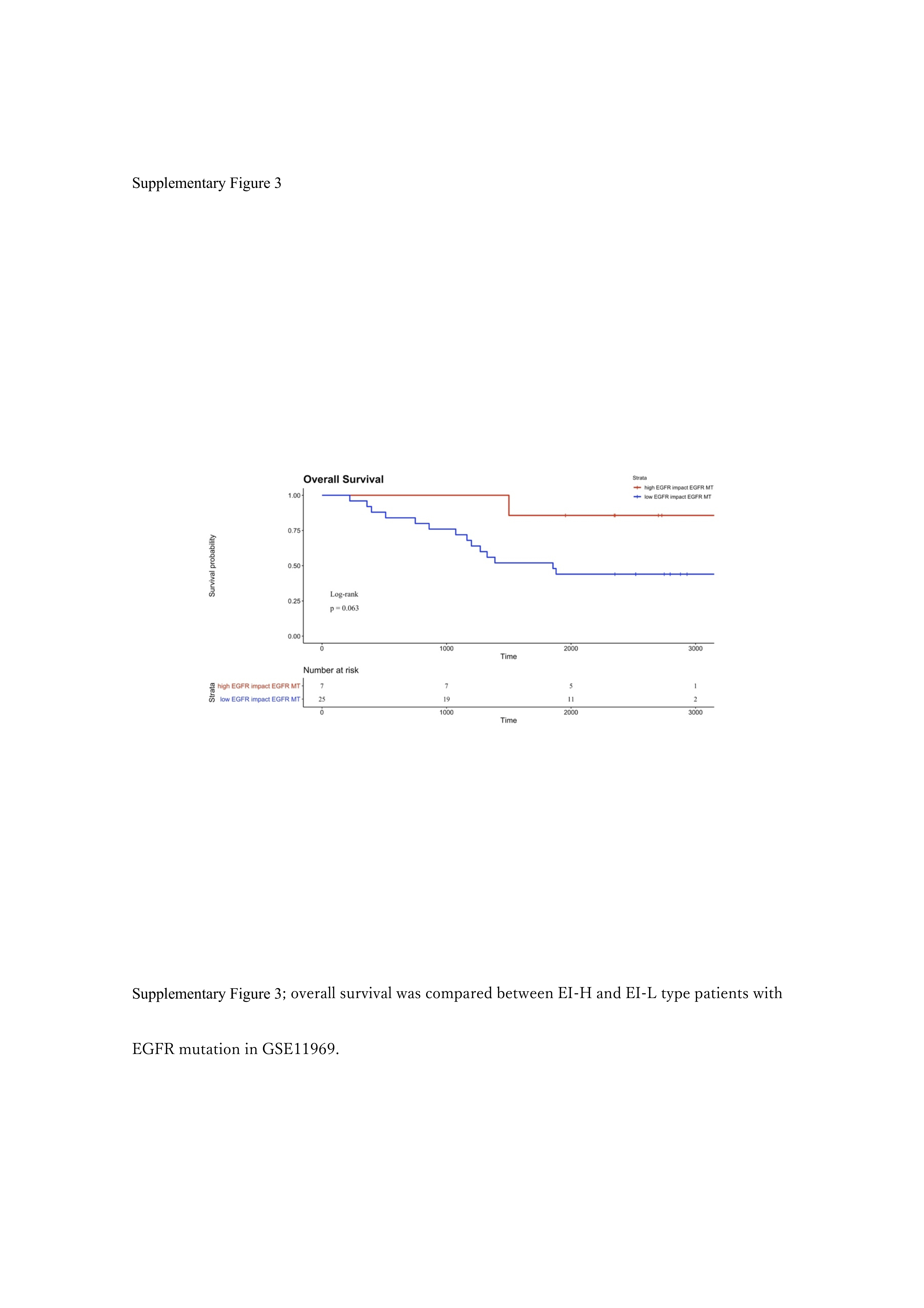

Supplement: Supplementary file 3 — Supplementary Figure 3. [file 41598_2020_63200_MOESM3_ESM.jpg]
