# Supplementary material for: A novel diagnostic system to evaluate epidermal growth factor receptor impact as a prognostic and therapeutic indicator for lung adenocarcinoma
Source: Sci Rep. 2020 Apr 10;10:6214. doi: 10.1038/s41598-020-63200-7 (PMC7148318; doi:10.1038/s41598-020-63200-7)
Supplement: Supplementary file 4 — Supplementary information. [file 41598_2020_63200_MOESM4_ESM.docx]

Title: A novel diagnostic system to evaluate epidermal growth factor receptor impact as a prognostic and therapeutic indicator for lung adenocarcinoma

Authors: Kazuya Takakuwa^a^, Kaoru Mogushi^b^, Min Han^b^, Tomoaki Fujii^c^, Masaki Hosoya^b^, Arina Yamanami^d^, Tomomi Akita^a^, Chikamasa Yamashita^a^, Tetsu Hayashida^e^, Shunsuke Kato^b^, and Shigeo Yamaguchi^b^,*

^a^Department of Pharmaceutics and Drug Delivery, Faculty of Pharmaceutical Sciences, Tokyo University of Science, Yamazaki, Noda, Chiba, Japan

^b^Department of Clinical Oncology, Juntendo University Graduate School of Medicine, Hongo, Bunkyo-ku, Tokyo, Japan

^c^Department of Cancer Genome Research, Sasaki Institute, Sasaki Foundation, Kandasurugadai, Chiyoda-ku, Tokyo, Japan

^d^International School of the Sacred Heart, Shibuya-Ku, Tokyo, Japan

^e^Department of Surgery, Keio University School of Medicine, Shinanomachi 35, Shinjuku-ku, Tokyo, Japan

*Corresponding author: Shigeo Yamaguchi, Department of Clinical Oncology, Juntendo University Graduate School of Medicine, Hongo, Bunkyo-ku,, Tokyo 113-8421, Japan, Phone No: +81-3-5802-1543, Fax No: +81-3-5684-8035, Email Address: [yamashige33@gmail.com](mailto:yamashige33@gmail.com)
